# uPAR-targeted multimodal tracer for pre- and intraoperative imaging in cancer surgery

**DOI:** 10.18632/oncotarget.3680

**Published:** 2015-03-29

**Authors:** Martin C. Boonstra, Pieter B.A.A. van Driel, Danny M. van Willigen, Marieke A. Stammes, Hendrica A.J.M. Prevoo, Quirijn R.J.G. Tummers, Andrew P. Mazar, Freek J. Beekman, Peter J.K. Kuppen, Cornelis J.H. van de Velde, Clemens W.G.M. Löwik, John V. Frangioni, Fijs W.B. van Leeuwen, Cornelis F.M. Sier, Alexander L. Vahrmeijer

**Affiliations:** ^1^ Department of Surgery, Leiden University Medical Center, Leiden, Netherlands; ^2^ Department of Radiology, Leiden University Medical Center, Leiden, Netherlands; ^3^ Percuros BV, Enschede, Netherlands; ^4^ Department of Chemistry of Life Processes Institute, Northwestern University, Evanston, IL, USA; ^5^ Robert H. Lurie Comprehensive Cancer Center, Northwestern University, Evanston, IL, USA; ^6^ MILabs, Utrecht, Netherlands; ^7^ Section Radiation, Detection and Medical Imaging, Delft University of Technology, Delft, Netherlands; ^8^ Curadel, LLC, Worcester, MA, USA; ^9^ Antibodies for Research Applications BV, Gouda, Netherlands

**Keywords:** Image-guided surgery, near-infrared, SPECT, dual labeling, colorectal

## Abstract

Pre- and intraoperative diagnostic techniques facilitating tumor staging are of paramount importance in colorectal cancer surgery. The urokinase receptor (uPAR) plays an important role in the development of cancer, tumor invasion, angiogenesis, and metastasis and over-expression is found in the majority of carcinomas. This study aims to develop the first clinically relevant anti-uPAR antibody-based imaging agent that combines nuclear (^111^In) and real-time near-infrared (NIR) fluorescent imaging (ZW800-1). Conjugation and binding capacities were investigated and validated *in vitro* using spectrophotometry and cell-based assays. *In vivo,* three human colorectal xenograft models were used including an orthotopic peritoneal carcinomatosis model to image small tumors. Nuclear and NIR fluorescent signals showed clear tumor delineation between 24h and 72h post-injection, with highest tumor-to-background ratios of 5.0 ± 1.3 at 72h using fluorescence and 4.2 ± 0.1 at 24h with radioactivity. 1-2 mm sized tumors could be clearly recognized by their fluorescent rim. This study showed the feasibility of an uPAR-recognizing multimodal agent to visualize tumors during image-guided resections using NIR fluorescence, whereas its nuclear component assisted in the pre-operative non-invasive recognition of tumors using SPECT imaging. This strategy can assist in surgical planning and subsequent precision surgery to reduce the number of incomplete resections.

## INTRODUCTION

Diagnosis, staging, and surgical planning of colorectal cancer patients increasingly rely on imaging techniques that provide information about tumor biology and anatomical structures [1-3]. Single-photon emission computed tomography (SPECT) and positron emission tomography (PET) are preoperative nuclear imaging modalities used to provide insights into tumor location, tumor biology, and the surrounding micro-environment [4]. Both techniques depend on the recognition of tumor cells using radioactive ligands. Various monoclonal antibodies, initially developed as therapeutic agents (e.g. cetuximab, bevacizumab, labetuzumab), are labeled with radioactive tracers and evaluated for pre-operative imaging purposes [5-9]. Despite these techniques, during surgery the surgeons still rely mostly on their eyes and hands to distinguish healthy from malignant tissues, resulting in incomplete resections or unnecessary tissue removal in up to 27% of rectal cancer patients [10, 11]. Incomplete resections (R1) are shown to be a strong predictor of development of distant metastasis, local recurrence, and decreased survival of colorectal cancer patients [11, 12].

Fluorescence-guided surgery (FGS) is an intraoperative imaging technique already introduced and validated in the clinic for sentinel lymph node (SLN) mapping and biliary imaging [13]. Tumor-specific FGS can be regarded as an extension of SPECT/PET, using fluorophores instead of radioactive labels conjugated to tumor-specific ligands, but with higher spatial resolution than SPECT/PET imaging and real-time anatomical feedback [14]. A powerful synergy can be achieved when nuclear and fluorescent imaging modalities are combined, extending the nuclear diagnostic images with real-time intraoperative imaging. This combination can lead to improved diagnosis and management by integrating pre-, intra- and postoperative imaging. Nuclear imaging enables pre-operative evaluation of tumor spread while during surgery deeper lying spots can be localized using the gamma probe counter. The (NIR) fluorescent signal aids the surgeon in providing real-time anatomical feedback to accurately recognize and resect malignant tissues. Postoperative, malignant cells can be recognized using NIR fluorescent microscopy. Clinically, the advantages of multimodal agents in image-guided surgery have been shown in patients with melanoma and prostate cancer, but those studies used a-specific agents, following the natural lymph drainage pattern of colloidal tracers after peri-tumoral injection [15, 16].

The urokinase-type plasminogen activator receptor (uPAR) is implicated in many aspects of tumor growth and (micro) metastasis [17, 18]. The levels of uPAR are undetectable in normal tissues except for occasional macrophages and granulocytes in the uterus, thymus, kidneys and spleen [19]. Enhanced tumor levels of uPAR and its circulating form (suPAR) are independent prognostic markers for overall survival in colorectal cancer patients [20, 21]. The relatively selective and high overexpression of uPAR in a wide range of human cancers including colorectal, breast, and pancreas nominate uPAR as a widely applicable and potent molecular target [17, 22].

The current study aims to develop a clinically relevant uPAR-specific multimodal agent that can be used to visualize tumors pre- and intraoperatively after a single injection. We combined the ^111^Indium isotope with NIR fluorophore ZW800-1 using a hybrid linker to an uPAR specific monoclonal antibody (ATN-658) and evaluated its performance using a pre-clinical SPECT system (U-SPECT-II) and a clinically-applied NIR fluorescence camera system (FLARE™).

## RESULTS

### Conjugation and specificity

**Figure 1:**
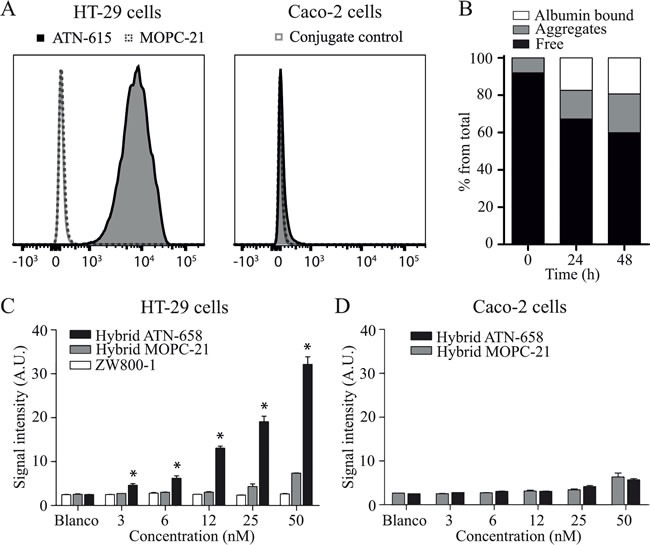
*In vitro* agent validation **A**) Flow cytometer analyses show high uPAR expression on HT-29 cells while no expression is detectable on the Caco-2 cell line. **B**) Graph shows the serum stability of hybrid ATN-658. An increase in aggregates and albumin bound agents is seen over time, with 60% of the agent still free after 48 h. **C**) Cell based plate assay analyses show the specific binding of hybrid ATN-658 on uPAR expressing HT-29 cells. Hybrid ATN-658 signal intensities differed significantly from the control hybrid MOPC-21 at all dose groups except 0 nM. **D**) No specific binding on the control cell line Caco-2 and there were no significant differences between both tracers at all dose groups. A.U.= arbitrary units.

uPAR was confirmed to be expressed on HT-29 colorectal cancer cells with around 20,000 copies per cell, which is considered moderate compared to previously reported values between 50,000-200,000 on monocytoid cells and neo-angiogenic endothelial cells. Caco-2 colorectal cancer cells showed minimal expression (<1000 copies per cell) and was used as a control cell line (Figure 1A). ATN-658 and isotype antibody control MOPC-21 were conjugated to the hybrid label (DTPA-Lys(ZW800)-Cys-NHS) in mean ratios (dye:antibody) of 1.7:1 and 2.2:1 respectively. Cell based plate assay analyses were performed to evaluate retained binding capacity of the agents after conjugation. On HT-29 cells a dose-dependent fluorescent signal was detected with hybrid ATN-658, whereas with hybrid MOPC-21 no specific signals were obtained, except at the highest concentrations (Figure 1C). Single NIR dye ZW800-1 showed no signals at all. As expected, on the Caco-2 cells no specific signals were observed with either hybrid ATN-658 or hybrid MOPC-21 (Figure 1D). HPLC analysis showed that hybrid ATN-658 monoclonal antibody was moderately stable in human serum: 60% of the agent was still free after 48h, while the remaining 40% was aggregated or bound to serum albumin (Figure 1B).

### Nuclear imaging using SPECT and bio-distribution

**Figure 2:**
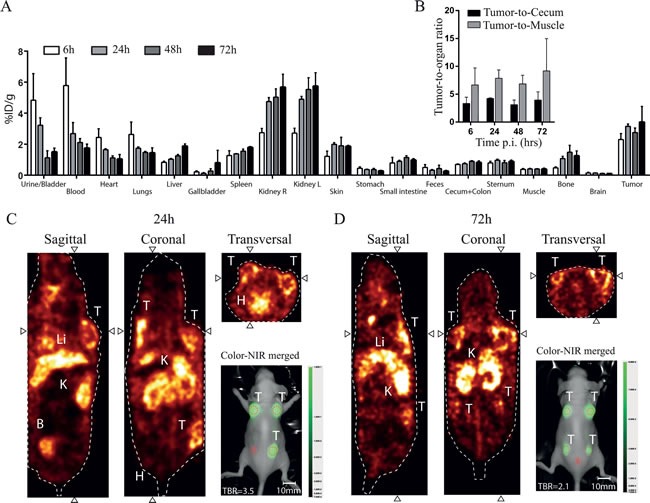
Biodistribution pattern of hybrid ATN-658 **A**) Biodistribution data of hybrid ATN-658 in the HT-29 subcutaneous colorectal model in mice. Graph shows increased tumor uptake over time and decreasing signals in the urine, blood, heart and lungs, as determined by a gamma counter and represented as %ID/weight. **B**) Mean tumor-to-cecum and tumor-to-muscle ratios over time up to 72 h post injection are shown including standard deviations. Images show SPECT scans from **C**) 24 h (mouse with 3 tumors) and **D**) 72 h (mouse with 4 tumors) post injection, revealing the broad imaging window. Activity was seen in the tumors and the metabolizing organs (kidneys and liver). Inserted are representative NIR fluorescent images taken subsequent to SPECT imaging with the pre-clinical PEARL system at 24h and 72h post injection, showing the usability of the multimodal agent. Red dotted circles = Regions of interest (with B as Background), T= Tumor, Li= Liver, K= Kidney, B= Bladder, H=Heart.

After 6, 24, 48 and 72 hours, SPECT imaging and biodistribution studies were performed in the subcutaneous HT-29 colorectal cancer model in mice. Mice were injected with 150 μg (1 nmol) hybrid ATN-658 conjugated to ^111^In with activities for mice measured and sacrificed at 6 h post injection of 32.6 ± 0.1, at 24 h 33.1 ± 0.7, at 48 h 32.8 ± 0.9 and at 72 h 34.0 ± 1.2 (MBq, mean ± SD). The biodistribution study using SPECT and gamma-counter confirmed accumulation of hybrid ATN-658 in subcutaneous colorectal tumors and metabolizing organs. The bio-distribution pattern and kinetics showed high percentages in urine, blood, heart and lungs at 6 h, which decreased over time due to clearance as indicated by the increasing signals in the kidneys and liver (Figure 2A). High signals in the skin were observed compared to the signals from the intestine, influencing TBRs, as also seen with NIR fluorescence in this subcutaneous model. Using the gamma counter, the tumor-to-colon (Figure 2B) ratios of mice that received hybrid ATN-658 were 3.4 ± 0.9, 4.2 ± 0.1, 3.1 ± 0.7 and 4.0 ± 1.2 at 6 h, 24 h, 48 h and 72 h respectively. While the tumor-to-muscle ratio (Figure 2B) was higher: 6.7 ± 2.5, 7.9 ± 1.2, 6.9 ± 1.3 and 9.2 ± 4.72 respectively at the same time points. On the basis of these results, an optimal imaging window between 24 and 72h was established. The presence in the tumors of the agent was stable over time. Figure 2C shows examples of the SPECT images indicating signals in the tumor, liver, kidney and bladder at 24 h. After 72 h (Figure 2D) the radioactive signal in the tumors could still be clearly recognized, but also signals in the liver and kidneys were present. The SPECT images were not interpreted quantitatively. Simultaneously acquired fluorescence images confirmed the tumor specific accumulation of hybrid ATN-658 (Figure 2C and 2D).

### *In vivo* binding characteristics and dose optimization

**Figure 3:**
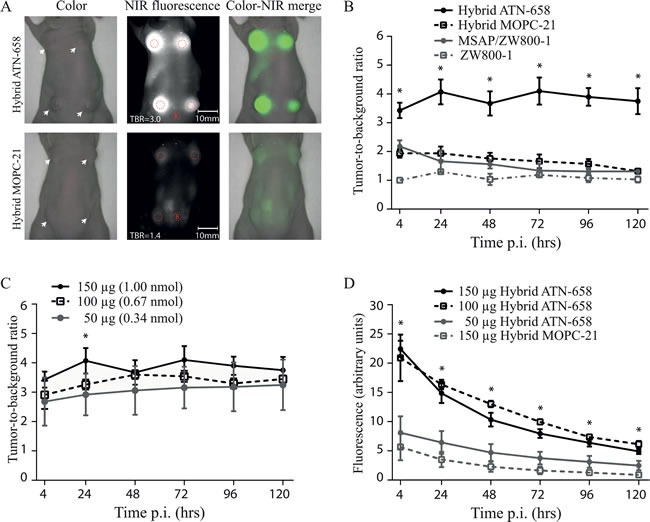
*In vivo* agent validation using the subcutaneous colorectal model **A**) UPPER ROW: The images show representative fluorescent signals in a mouse injected with 150 μg/1 nmol of hybrid ATN-658 and measured at 24 h post injection with a mean TBR of 4.1. White arrows indicate tumors. LOWER ROW: Mouse injected with 150 μg/1 nmol of hybrid MOPC-21 with a mean TBR of 1.6. White arrows indicate tumors. Red dotted circles = Regions of interest (with B as Background). **B**) TBRs over time up-to 120h post injection of mice receiving 1 nmol of the different agents. At each time point, at least 3 mice for each group were measured. Hybrid ATN-658 TBRs were significantly different from all three controls at all time-points. **C**) TBRs of various doses of hybrid ATN-658. Overall there were no significant differences between the TBRs of all three dose groups except at 24 h between 50 ug and 150 ug (p=0.05). Furthermore, a decreasing trend in TBR was seen in the lower dose groups. **D**) Absolute signals from the different dose groups and agents in the tumors. Significant differences were seen between the lowest dose group (50 μg/0.34 nmol), the control agent (15 0μg/1 nmol) and the two highest uPAR specific dose groups (100 μg/0.67 nmol and 150 μg/1 nmol) at all time points. Between the 150 μg hybrid ATN-658 and 100 μg hybrid ATN-658 were no significant differences found. A.U.= arbitrary units

Subcutaneous HT-29 tumor bearing mice were intravenously injected for NIR fluorescent measurements with non-radioactive hybrid ATN-658, hybrid MOPC-21, DTPA-Lys(ZW800)Cys-NH_2_ or ZW800-1 alone in doses based on the nuclear imaging study. Using hybrid ATN-658, tumors could clearly be recognized in the subcutaneous tumor model (Figure 3A) from 24 till 72h post injection with doses ranging from 50-150 μg per mouse (Figure 3B and 3C), while the signals from the control antibody were barely visible. The uPAR specific probe resulted in stable TBRs at all time points (mean 3.9 ± 0.2), while the TBRs from control agents were significantly lower and decreasing over time towards the level of injections with the fluorophore ZW800-1 alone (Figure 3B). Although the absolute signal decreased significantly with decreasing doses (Figure 3D), no significant reduction in TBRs was observed. The lowest dose (50 μg; 0.34 nmol) showed slightly higher absolute signals when compared to 150 μg (1 nmol) of the control compound.

### NIR fluorescence in orthotopic models

**Figure 4:**
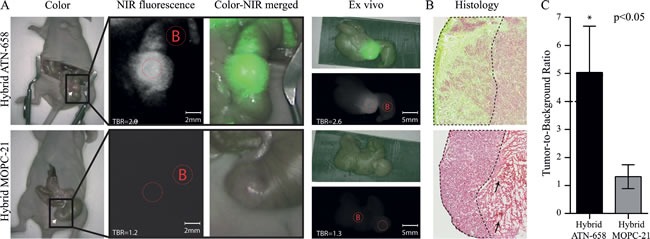
*In vivo* images and TBRs of mice bearing human orthotopic tumors **A**) UPPER ROW: Representative images of a mouse injected with 75 μg/0.5 nmol of hybrid ATN-658. The tumor on the cecum is exposed using a laparotomy, and measured after 72 h. Fluorescent signals in the colon surrounding the tumor are due to ingested agents as the signals is disappeared when the colon is emptied as seen on the *ex vivo* images. White arrow indicate the tumor. LOWER ROW: Mouse injected with 75 μg/0.5 nmol hybrid MOPC-21 which showed no specific NIR fluorescent signals. White arrow indicate the tumor. Red dotted circles = Regions of interests (with B as Background). **B**) Merged images (NIR fluorescent microscopy and histology) show co-localization of the uPAR specific multimodal agent, especially at the tumor border (border surrounded with dashed line). Apart from minor fluorescent signals in necrotic areas (black arrow), no tumor-specific signals are seen with the control agent. (magnification 40x) **C**) Graph shows the mean TBR and SD (n=3 for each group) of mice with orthotopic colorectal tumors after 72 h (*P* < 0.05).

Based on the NIR fluorescent results and the dose finding experiment from the subcutaneous colorectal model, the 72h post-injection time point in combination with the 0.5 nmol dose was chosen for the orthotopic models. Figure 4A shows typical examples of the orthotopic colorectal model. One clear fluorescent spot is shown in the mouse with the uPAR specific agent after exploration of the abdominal cavity, while no signals are measured in the mouse with the control probe. Some background signals were observed in the cecum as a result of ingestion of the agent, as the signals disappeared when the cecum was emptied as seen on the *ex vivo* images. *Ex vivo* fluorescence measurements validated the tumor specific location of the agent. Clear histological co-localization is shown between tumor cells and the NIR fluorescent signal in the uPAR specific group and no tumor specific signals were seen in the control tumors (Figure 4B). For the orthotopic colorectal model, a mean NIR fluorescent TBR of 5.0 ± 1.3 was measured with the uPAR specific hybrid agent while the control agent showed a mean TBR of 1.3 ± 0.3 (Figure 4C).

**Figure 5:**
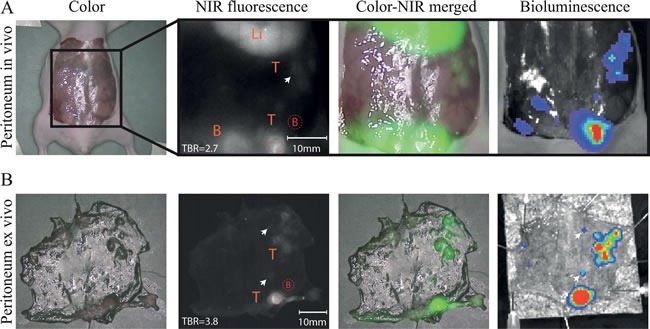
*In vivo* peritoneal carcinomatosis model **A**) ***In vivo*** images showed fluorescent rims around different sizes of peritoneal tumors/ metastasis (white arrow) which are validated using bioluminescence. Li= Liver, B= Bladder, T=Tumors. **B**) *Ex vivo* images of the peritoneal tumors/metastases confirmed the rim shaped signals around the tumors. T= Tumors. Arrows indicate very small metastases (≤1-2mm) which were fluorescently delineated. Red dotted circles = Regions of interest (with B as Background).

The peritoneal carcinomatosis model confirmed the ability of the uPAR specific agent to visualize small metastases between 1-2 mm in size. Clear fluorescent spots were recognized in the peritoneum even next to high background signals from the liver and the bladder (Figure 5A). BLI imaging validated these spots to be malignant cells. *Ex vivo* images showed enhanced demarcation of small tumors/metastases due to absence of the background signals from the bladder and the liver (Figure 5B) and enabled the recognition of extra tumors (white arrowheads). While primary tumors showed homogeneous signals, the smaller metastases were characterized by a rim staining.

## DISCUSSION

Not all tumor-associated biomarkers are suitable as oncotarget for therapy or imaging. If these proteins are homogeneously expressed on the cellular membrane in at least 10-times higher densities than on the surrounding normal cells, than they are considered potential candidates as imaging target [23]. uPAR is an oncotarget, which primary function is to focus the proteolytic effect of its ligand urokinase-type plasminogen activator (uPA), enhancing the migration and invasion capacities of tumors [24]. High endogenous levels of uPAR are found in invasive borders and hypoxic regions of solid tumors resulting in highly specific and sensitive targeting [25]. We previously investigated the presence of uPAR in tumors from 262 colorectal cancer patients [22]. The majority (85%) of these tumors showed uPAR expression not only on malignant cells, but also in tumor-associated stromal cells (such as tumor-associated macrophages), which was negatively associated with overall-survival and disease-free-survival [22]. This expression pattern is also seen in other studies investigating colorectal and breast cancer [26, 27]. The simultaneous targeting of tumor and tumor surrounding stromal cells increases the percentage of tumor mass that will be targeted. The additional effect of stromal cells is not reflected by the models used in this study, because the antibody recognizes only human uPAR, that is present on the relatively low uPAR-expressing HT-29 cells. Therefore, due to the combined expression on tumor cells and tumor-associated stromal cells, higher absolute signals (and TBRs) can be expected when applied in humans.

This study reports the development and feasibility of an uPAR recognizing multimodal agent that was evaluated for SPECT and NIR fluorescent imaging after a single low dose injection. The biodistribution analysis showed a classical antibody distribution pattern with decreasing signals in urine, blood, heart and lungs over time, culminating in increasing signals in the liver due to metabolization and clearance of the agent. The relatively high signals in the kidneys compared to liver and tumor tissue are remarkable because the majority of (monoclonal) antibodies are retained and cleared via the liver rather than the kidneys [28]. This phenomenon was equally present in the specific as well as in the control conjugate and was noticeable with both the radioactive label and NIRF-dye (data not shown). This phenomenon could be due to a suboptimal purification of the conjugates, leaving free hybrid label in the circulation. However, this would have been cleared from the circulation within several hours, unless if this label aggregated with serum proteins like albumin. Another explanation could be the dissociation of (part of) the conjugates, although this has not been reported before with this hybrid molecule. The kidney accumulation has not disrupted the results of this preclinical evaluation, but this issue should be resolved with respect to clinical studies.

Between 24 and 72 h, tumors could be clearly visualized with both SPECT and NIR fluorescent imaging. Because nuclear and NIR fluorescent tumor-to-background ratios were comparable over time the following *in vivo* experiments were performed solely with the NIR fluorescent label, decreasing the amount of required nuclear label. Especially the TBRs calculated with the cecum as background are clinical relevant and, although lower than the tumor-to-muscle ratios, these ratios were more than sufficient to adequately recognize the tumors. The agent can be administered in the nanomolar range and visualized sub-millimeter sized tumors/ metastasis, which were otherwise invisible to the human eye. This is especially clinically important for colorectal cancer patients with peritoneal carcinomatosis as these lesions are generally small, difficult to distinguish from adjacent normal tissue and numerous. Furthermore, the amount of tumor reduction is directly related to overall survival [29]. The difference in fluorescent TBRs found between the subcutaneous and orthotopic colorectal model can be explained by the relatively high signals measured in the skin compared with the signal in the intestine. No differences in absolute fluorescent signals were seen between the 100 μg (0.67 nmol) and the 150 μg (1.00 nmol) dose group, possibly due to saturation of the receptors. The inclusion of non-specific controls showed the agent-specific origin of the signals and excluded signals caused by the so-called enhanced permeability and retention effect [30-34]. For further clinical translation it is important to perform a dose escalation study, as decreasing the dose will lead to a decrease in possible adverse events.

Earlier studies already indicated the importance of multimodal agents integrating pre-operative SPECT and intraoperative NIRF fluorescent imaging. Li et al. conjugated the peptide cRGD to IRDye800CW and ^111^In and showed its ability to specifically bind αvβ3 in human melanoma xenografts. But due to the fast blood clearance, a small imaging window and relatively low signals were observed [35]. Bunschoten et al. also developed a RGD based hybrid derivate using the same multimodal linker as used in this study, showing prolonged retention and increased tumor accumulation compared to the more conventional DTPA based agents [36]. Two studies using identical dual-labels (^111^In-DTPA-IRDye800CW) conjugated to either anti-CEA or anti-PSMA specific antibodies described large imaging windows (2-3 days) in colorectal and prostate xenograft tumor models [37, 38]. Although these studies are not easily comparable because they used different models and different targeting agents, the results might indicate a possibly size-related advantage of antibodies over peptide-based agents, as predicted earlier by Wittrup et al. [39]. They mention that IgG-based conjugates exhibit the most favorable balance between systemic clearance and vascular extravasation, resulting in maximal tumor uptake and subsequent optimal tumor specific signals.

The generally accepted limit for cell detection with 2D optical imaging systems lies between 10^1^-10^4^ cells/cm^2^ [2], which is in the range of the nodules in our peritoneal carcinomatosis model. These nodules were remarkably visible, considering their number of cells, but showed a fluorescent rim rather than a homogenous signal as was observed by larger tumors. The rim effect could be due to the high affinity of the agent, as it is known to be an influencing factor for agent distribution throughout small tumor spheroids, or due to absence of (neoangiogenic) vasculature [40, 41]. Although HT-29 cells express only moderate levels of uPAR, it has been reported that dormant micro-metastasis specifically up-regulate uPAR on their cellular membrane [42]. So, the relatively high signals found in our HT-29 model suggest that uPAR might especially be suited for the detection/visualization of very small tumors/metastasis, as are frequently seen in the peritoneum of colorectal cancer patients [29, 43].

Although several uPAR directed agents are conjugated to (NIR) fluorophores, such as Cy5.5 [44] and IRDye800CW [45], isotopes [46, 47] and nanoparticles [45, 48, 49] these probes are only pre-clinically evaluated and no uPAR specific molecular imaging agents are available for the clinic yet. Ligands utilized are small peptides (MW 1-2kd), the amino-terminal fragment (ATF) of urokinase (MW 20kd) or monoclonal antibodies (MW 150kd), all directed to the extracellular region of uPAR. In a syngeneic model, the murine ATF conjugated to a NIR dye showed clear tumor accumulation after 24 h aiding in the recognition of tumor margins up-to 13 days [45]. The ^64^Cu-labeled human uPAR binding peptide DOTA-AE105 showed high affinity for uPAR and possessed rapid and high tumor accumulation capabilities [47, 50]. DOTA-AE105 was also radiolabeled with ^177^Lu creating local tumor radiation, eliciting significant decreases in tumor size [51]. Both agents show promising imaging and therapeutic results, but due to their small size and short imaging window they are less suitable for multimodal imaging approaches. Furthermore, it is shown that functional groups can alter the binding characteristics, pharmacokinetics, and dynamics of smaller targeting determinants like peptides and fragments of antibodies considerably, resulting in faster blood clearance and increased liver accumulation, and hampering a clinical translation [52-54]. We used the full-size antibody ATN-658 to minimize these effects and to facilitate a relatively easy clinical translation, as a humanized version is already available for clinical studies [55]. ATN-658 binds to uPAR regardless whether urokinase (uPA) is bound or not, offering a clear advantages over the frequently used previously mentioned ATF of uPA or other anti-uPAR antibodies [56]. Recently LeBeau et al. described imaging results using an uPAR-specific antibody interacting with uPA binding, with both SPECT/CT and optical imaging. Although the data were very convincing, this ‘proof-of-concept’ study used clinically not applicable antibodies, fluorophores and imaging systems [56]. ATN-658 can bind to a residual fragment of uPAR frequently observed in tumors that remains attached to the membrane after proteolysis [57]. With respect to safety issues and adverse effects, ATN-658 does not internalize, minimizing its effect on the processes within the cells [58]. Furthermore, due to the single injection needed for imaging applications and the low doses of hybrid ATN-658 needed, no or minimal side effects are expected when introduced in humans. The dye ZW800-1 used in this study is a functionalized NIR fluorescent (800 nm) zwitterionic fluorophore with optimal *in vivo* characteristics such as low background signal, fast body clearance, high quantum yield, low light scattering, and a full toxicology report present [59]. ZW800-1 is available as a cGMP grade product and can consequently be applied in humans. The combination of a humanized antibody with a cGMP grade dye as used in this study should propagate a fast clinical translation.

To avoid over-interpretation of pre-clinical data it is important to evaluate novel imaging agents with both pre-clinical imaging systems (as reference system) as well as clinically-compatible systems. The use of closed box chambers, shielding residual light, in combination with optimized exposure times, as generally used for pre-clinical evaluations are conditions that cannot be met clinically. Therefore, the uPAR specific multimodal agent in this study was visualized with both pre-clinical as clinically applied imaging systems.

In conclusion, combining FGS with conventional 3D nuclear techniques in one single agent overthrows the limitations of optical imaging in being a surface technique with maximal 10 millimeters depth penetration. We developed the first clinical relevant antibody-based uPAR-specific multimodal agent combining both NIR fluorescence and nuclear imaging. As uPAR is up-regulated on many cancer types, the novel agent can be applied for broad indications. The clinical relevant settings in this study regarding doses, antibodies, fluorophores, a single injection and the clinically-compatible imaging system should ensure a relatively conditioned clinical translation of this uPAR recognizing multimodal agent.

## MATERIALS AND METHODS

### Cell lines

The human colorectal adenocarcinoma cell lines HT-29 and Caco-2 were cultured in respectively RPMI-1640 and DMEM (Invitrogen, Bleiswijk, Netherlands). Both media were supplemented with 10% Fetal Clone II (FCII; Hyclone, Thermo Scientific, Rockford, IL, USA), 100 IU/ml penicillin and 100 IU/ml streptomycin (Invitrogen). Both cell lines were grown in a humidified incubator at 37°C and 5% CO_2_. Cell culture medium was replenished every second day. The HT-29/luc2 cell line was established as described before and cultured under the same conditions [60].

### Monoclonal antibodies

Mouse monoclonal antibodies ATN-615 and ATN-658 are both of the IgG_1k_ isotype and bind with high affinity (Kd ≈ 1nM) to epitopes on domain D3 of uPAR and are extensively validated for *in vitro* and *in vivo* applications [61, 62]. ATN-615 is optimized for *in vitro* experiments and ATN-658 for *in vivo*. The control monoclonal IgG_1k_ isotype MOPC-21 was purchased from BioXCell (West Lebanon, USA) and has an no known specificity after testing on human and rodent tissues.

### Flow cytometry

Flow cytometry was used to evaluate uPAR expression on HT-29 (stage II carcinoma, moderate uPAR expression) and Caco-2 (stage II carcinoma, low uPAR expression) cells. Cells were grown to 90% confluence and detached with trypsin /EDTA. After evaluation of viability using trypan blue, cells were adjusted to 0.5 × 10^6^ cells/tube in ice cold PBS and incubated with 100 μl anti-uPAR antibody or non-specific control on ice for 30 minutes. After incubation, cells were washed three times in ice-cold PBS and incubated with a goat anti-mouse IgG_1_–AF488 secondary antibody (A21121, Life Technologies, 1/800) on ice for 30 minutes. After three washing steps with ice cold PBS, cells were re-suspended in 400 μl PBS containing propidium iodide to stain dead cells. Samples were measured on a LSRII flow cytometer (BD Biosciences). Per sample ten thousand living cells were counted. Furthermore, flow cytometry was used to evaluate the actual number of uPAR specific antibodies present on single HT-29 cells using the Qifi-kit (Dako, Denmark). A standard curve was generated using calibration beads. The antibody binding capacity was distracted from this standard curve after which the actual number of bound antibodies could be calculated and corrected for the isotype control MOPC-21.

### Multimodal conjugation

ATN-658 and MOPC-21 were conjugated to the zwitterionic fluorophore ZW800-1 (λ_ex_=773 nm, λ_em_= 790 nm) and radiolabeled with ^111^In using a hybrid label called MSAP (multifunctional single attachment point) [59, 63]. The hybrid label (DTPA-Lys(ZW800)-Cys-NHS) was synthesized according to previously described procedures [59] with the following deviations: Pyridine and DMSO were used to conjugate ZW800-NHS. A stock solution (DMSO) with a concentration of 5.14 mM was prepared.

For the uPAR specific hybrid agent (DTPA-Lys(ZW800)-Cys-ATN-658), 300 μl of phosphate buffer pH 8.4 was added to 18.4 nmol of ATN-658 and 36 μl of the hybrid label stock solution (185 nmol, 10 eq). The mixture was stirred at room temperature for 3h. Excess of hybrid label was removed using a 7K Zeba Spin desalting column (Thermo Scientific, Rockford, IL, USA). For the control hybrid agent (DTPA-Lys(ZW800)-Cys-MOPC-21), 100 μl of phosphate buffer pH 8.4 was added to 6.7 nmol of MOPC-21 and 33 μl of the hybrid label stock solution (168 nmol, 25 eq) and the mixture was stirred at room temperature for 3h. Excess of hybrid label was removed using a 7K Zeba Spin desalting column (Thermo Scientific, Rockford, IL, USA). This resulted in 4 different agents: hybrid ATN-658, hybrid MOPC-21, hybrid label (DTPA-Lys(ZW800)-Cys-NH2) and fluorophore alone (ZW800-1). Dye-to-antibody ratios were determined by measuring the absorbance at 280 nm (antibody) and 773 nm (dye) using a spectrophotometer (Pharmacia Biotech, Ultrospec 3000). Ratios were computed as follows: first, the concentration was calculated by dividing the fluorescent signal over the extinction coefficient (dye 249,000 M^−1^ cm^−1^ and antibody 225,000 M^−1^ cm^−1^) and multiplied by 10^6^. Secondly, concentrations were divided by each other, which resulted in the labeling ratio.

### Agent stability in serum

Human serum and dissolved sodium azide were filtrated using a 2.22 μm filter. Hybrid ATN-658 was added to a 24-wells plate, which was prepared with 0.02% sodium azide, in an agent:serum ratio of one. As control, serum was replaced by PBS. The 24-wells plate was incubated at 37°C with 5% CO_2_. HPLC analysis was performed using a size exclusion protein column (Phenomenex, USA) at 0, 24 h and 48 h with a flow-rate of 0,5 ml/min for 60 minutes at 2 channels; 280 nm and 780nm for respectively the antibody and ZW800-1.

### Agent specificity

HT-29 and Caco-2 cells were plated in 96-wells plates at densities of 50.000 cells per well in 100 μl of culture medium. After 48h, cells were incubated for 1 hour at 37°C with different doses of hybrid ATN-658, hybrid MOPC-21 or ZW800-1 to evaluate the (retained) binding capacity of the antibodies. After antibody incubation, cells were washed two times with culture medium to discard excess non-bound agents. Fluorescent signals were imaged using an Odyssey scanner (focus offset 3 mm; 800-nm channel; intensity 10; LI-COR Biosciences, Lincoln, Nebraska). After imaging, cell membranes were permeabilized by incubation with acetone/methanol (40/60) for 10 minutes, followed by one washing step. The fluorescent signal was corrected for the number of cells using ToPro-3 (Invitrogen) nucleus staining. Cells were then incubated for 5 minutes at room temperature and washed twice. The fluorescent signals were imaged using the Odyssey scanner (focus offset 3mm; 700-nm channel; intensity 8). The experiments were performed in triplicate.

### Tumor mouse models

Six-week-old athymic female mice (CD1-Foxn1^nu^, Charles River Laboratories, l'Arbresle, France) weighing 25 - 35 gram received autoclaved normal pellet food and sterilized water ad libitum. Throughout tumor inoculation and imaging procedures, animals were anesthetized with 4% isoflurane for induction and with 2% isoflurane for maintenance with a flow of 0.5 L/min and were placed on a heated animal bed with an integrated nose mask. The Animal Welfare Committee of Leiden University Medical Center, the Netherlands, approved all animal experiments. All animals received humane care and maintenance in compliance with the “Code of Practice Use of Laboratory Animals in Cancer Research” (Inspection W&V, July 1999).

Three xenograft colorectal models were utilized: a subcutaneous colorectal model with HT-29 cells and two orthotopic models for colorectal cancer and peritoneal carcinomatosis using HT-29/luc2 cells. The cells were grown to 90% confluence; after trypsinization, cell viability was evaluated by trypan blue. To induce subcutaneous colorectal tumors, 5×10^5^ HT-29 cells in 50 μL RPMI1640 medium were injected at four sites at the back of the mice. Tumor growth was monitored longitudinally using a digital caliper. To induce orthotopic colorectal tumors, subcutaneously growing HT-29/luc2 colorectal tumors were harvested from the subcutaneous model and subsequently transplanted onto the cecum of mice as described by Tseng et al. [64]. Briefly, the cecal wall was slightly damaged to induce an immunoreaction and to facilitate tumor cell infiltration. Small tumor fragments (approximately 3 mm in diameter) were transplanted onto the cecal wall using a 6-0 suture. To induce peritoniteal carcinomatosis 1×10^6^ HT-29/luc2 cells (in 100 μL RPMI1640 medium) were injected into the abdominal cavity. Tumor growth of both orthotopic models was monitored twice a week by bioluminescence imaging (BLI), using an intraperitoneal injection of 150μg/g of D-luciferin solution (SynChem, Inc., Elk Grove Village, IL) in PBS, in a total volume of 50 μL, 10 minutes prior to imaging with the IVIS Spectrum imaging system (Caliper Life Sciences Inc., Hopkinton, MA, USA).

### SPECT and biodistribution

Radiolabeling was obtained by dissolving hybrid ATN-658 in 0.1M HEPES buffer (10μg/100μL) and adding indium (III)chloride (35MBq InCl_3_, Covidien-Mallinckrodt, Dublin, Ireland). After 30 min of incubation on the shaker the labeling was validated by HPLC, (JASCO, USA). In all cases, labeling efficacy was >90%. To study the biodistribution, 150 μg (1 nmol) of hybrid ATN-658 was intravascularly injected in 12 mice. SPECT scans were conducted at 6, 24, 48 and 72h post injection (3 mice for each group) with a 3-headed U-SPECT-II gamma camera (MILabs, Utrecht, the Netherlands).

The total body scan was acquired using a 0.6 mm mouse pinhole collimator with energy settings at 171 and 245 keV with a window of 20% and additional background windows of 4.5% [65]. Subsequently, images were reconstructed on 0.2×0.2×0.2mm voxels using 40 iterations POSEM [65]. A relatively low number of 2 subsets were chosen to prevent erasure of structures with extremely low activity [66]. A 3D Gaussian post filter with a Full Width of Half Max of 1.2 mm was used to suppress image noise. Images were processed using PMOD 3.6 software (Pmod Technologies Ltd, Zurich, Switzerland). On each time point, after the last imaging acquisition, mice (n=3) were sacrificed and organs were excised, weighted, and counted for radioactivity with a gamma counter (Wizard2 2470 automatic gamma scintillation counter, Perkin Elmer, USA). The %ID/weight was calculated as followed: (MBq measured in tissue/injected dose *100%) /weight of tissue.

### *In vivo* NIR fluorescence imaging

When the subcutaneous tumors were 36±6 mm^2^, either hybrid ATN-658 (50μg/0.34 nmol, 100 μg/0.67 nmol or 150 μg/1 nmol), hybrid MOPC-21 (150 μg/1 nmol), or controls DTPA-Lys(ZW800)-Cys-NH_2_ (1.83 μg/1 nmol) and ZW800-1 (1.15μg/1 nmol) were injected intravenously in 3 mice per group. NIR fluorescent signals were measured at time points 4, 24, 48, 72, 96, and 120h post-injection, using both the intraoperative *FLARE^TM^ system* [68] and the *PEARL small animal imaging system* (LI-COR Biosciences).

Mice bearing the orthotopic tumors underwent a laparotomy to evaluate tumor specific accumulation at 72h post injection. The injection was with either 75μg (0.5 nmol) hybrid ATN-658 (n=3 for both orthotopical models) or 75 μg (0.5 nmol) hybrid MOPC-21 (n=3, only orthotopic colorectal model). Regions of interest were selected and the images were normalized to control images after which intensity was computed using the imaging system associated software.

### Histological analysis

Tumors were surgically removed and either snap-frozen or paraffin embedded. Tumors were sectioned and scanned on the Odyssey Infrared Imaging System (LI-COR Biosciences, Lincoln, NE, USA), using the 800 nm channel, for evaluation of the fluorescent location. Subsequently, sections were stained with hematoxylin-eosin and merged images were generated to validate agent distribution and specificity.

### Statistical analysis

For statistical analysis and the generation of graphs GraphPad Prism software (version 5.01, GraphPad Software Inc, La Jolla, CA, USA) was used. Differences between groups in the binding assays were analyzed using the Mann-Whitney U test. Tumor-to-background ratios (TBR) were calculated using associated software or ImageJ by dividing the NIR fluorescent signal of the tumor by the NIR fluorescent signal of the surrounding tissue using respectively images from the PEARL small animal imager or FLARE system. TBRs are reported as mean and standard deviation. The two-way repeated measurement ANOVA, used to assess the relation between TBRs in the dose groups and time points, was corrected using the Bonferroni correction. P-values equal or lower than 0.05 were considered significant.
